# The influence of concomitant antiepileptic drugs on lamotrigine serum concentrations in Northwest Chinese Han population with epilepsy

**DOI:** 10.1371/journal.pone.0210600

**Published:** 2019-01-15

**Authors:** Xiaonian Han, Jing Huang, Jianhua Lv, Li Ma, Lirong Peng, Jinping Wang, Xiaojing Nie, Li Xia, Xin Zan

**Affiliations:** Department of Clinical Pharmacy, Xi’an Central Hospital, Xi’an, China; University of Catanzaro, ITALY

## Abstract

**Objective:**

The aims of this study were to identify the influencing factors such as gender, age, dose and combinations of other antiepileptic drugs (AEDs), especially in triple combinations on the pharmacokinetic of Lamotrigine (LTG) in epilepsy patients of Northwest Chinese Han population.

**Methods:**

Data of the LTG concentration and clinical information were analyzed retrospectively from a therapeutic drug monitoring (TDM) database at the Clinical Pharmacy Laboratory of Xi’an Central Hospital between January 1, 2016 and January 1, 2018. The independent-sample t-test, one-way ANOVA analysis and Bonferroni and Tamhane T3 *post-hoc* test, the stepwise multivariate regression analysis were adopted by IBM SPSS, version 22.0.

**Results:**

226 serum samples met the inclusion criteria and were evaluated. The mean LTG serum concentration was 5.48±3.83 μg/mL. There were no gender differences (P = 0.64), and there were no significant effects by age on LTG serum concentration after age stratification (3–14 years old, 14-45 years old, 45–59 years old) (P = 0.05). Multiple regression analysis showed that the daily LTG dose and co-administration of other AEDs significantly affected LTG serum concentrations. Combination with enzyme-inducer AEDs, the mean steady-state LTG concentration could be decreased by 30.73% compared with LTG monotherapy. Among enzyme-inducer AEDs, particularly strong inducer Carbamazepine (CBZ) could decrease the mean LTG concentration by 53.65%, but weak inducer AEDs such as Oxcarbazepine (OXC) and Topiramate (TPM) had no effect, Valproic acid (VPA) could increase the mean LTG concentration by 93.95%, and the inducer only partially compensated for the inhibitory effect of VPA in triple combination.

**Conclusions:**

There were no significant gender and age effects, but the LTG daily dose and co-administration of other AEDs significantly affected LTG serum concentration. Combination with enzyme-inducer AEDs, especially CBZ could significantly decrease LTG serum concentrations, VPA could significantly increase LTG serum concentrations, and the inducer only partially compensated for the inhibitory effect of VPA in triple combination. In the clinical setting, these findings can help to estimate LTG concentrations and adjust dosage and evaluate adverse drug reactions.

## Introduction

Lamotrigine(LTG) is a novel antiepileptic drug(AED), which is the first-choice drug for several epilepsy syndromes, such as focal and generalized epilepsy. LTG acts as a voltage-sensitive sodium channel inhibitor and also inhibits the release of excitatory amino acid neurotransmitters[1]. Oral absorption of the drug is rapid and complete with the peak concentration reached at about 1–3 h and an absolute bioavailability of 98%. Lamotrigine exhibits linear pharmacokinetics over a single-dose range of 50–400 mg[2,3]. LTG is principally metabolized at the 2-position of the triazine ring to form a quaternary ammonium glucuronide by uridine glucuronosyl transferease (UGT) 1A4 and UGT2B7 [4–6].

The LTG concentration is known to be influenced by gene polymorphisms and other combined AEDs. There are many reports about the effects of genetic polymorphisms on LTG concentrations [7–9], but little is known about the differences and the degree of influence on the pharmacokinetics of LTG in different ethnic population. Chang Y[10] reported that patients in Han Chinese of Northern China with the UGT1A4 142TT polymorphism had a higher blood LTG concentration and better therapeutic efficacy. Wang Q[11] reported that UGT1A4 genetic polymorphisms affected the serum lamotrigine concentrations in Chinese children with epilepsy. The drug interactions of LTG have been well described in double combination with one strong inducer or inhibitor[12–15], but most drugs were strong inducers such as phenobarbital, phenytoin sodium, which were seldom prescribed in our hospital and less prescribed in combination with LTG, and there was limited information about the influence of the weak inducer or triple combinations such as LTG+inducer+inhibitor. In our hospital, LTG is often prescribed with weak inducer or triple combination, so it is necessary to study the influence of AEDs on LTG concentration in our epilepsy patients of Northwest Chinese Han population.

The aims of this study were to evaluate the outcomes of TDM of lamotrigine in the Clinical Pharmacy Laboratory at Xi’an Central Hospital between January 1, 2016 and January 1, 2018, and identify the influencing factors such as gender, age, dose and combination of other AEDs, especially in triple combinations on the pharmacokinetics of LTG in epilepsy patients of Northwest China.

## Materials and methods

### Study patients

With the Medical ethics committee approval of Xi’an Central Hospital, we started the LTG serum concentration monitoring in epileptic patients Since January 2016. In this retrospective study, we collected the serum concentration data and clinical information of 226 patients who received LTG therapy alone or combined treatment and if they consented to be treated on the clinical setting and if they orally approved to use their information for epidemiological studies in the Clinical Pharmacy Laboratory at Xi’an Central Hospital between January 1, 2016 and January 1,2018, Inclusion criteria: the patients were older than 3 years and younger than 60 years, had taken a steady LTG dose for more than 1 month. Exclusion criteria: The people ≤ 3 years old because of a small number of patients and liver and kidney dysplasia, and the people ≥60 years old because of more diseases; Patients combined other diseases requiring medication treatment; serious hepatic or renal dysfunction; pregnant women and irregular drug users. Database collected from a well pre-designed table included: gender, age, height, body weight, LTG dose and course, combined medication, seizure control, time of blood sampling, time of last taking drug, and adverse drug reactions, et al. All data was anonymized before analysis.

### Blood samples and LTG concentration analysis

The steady-state LTG serum concentration was obtained after LTG had been maintained on the same dose for at least 1 month and other concomitant AEDs dose had no change during the preceding 1 month. Because of the potential fluctuation of LTG serum concentrations, this error of the data was minimized by only including blood samples that were collected just before the next dose. Blood samples treatment: Blood serum samples, adding internal standard barbiturate, were deposited down the protein with methanol, and then the supernatant was taken after centrifugation. The serum samples were analyzed by High Performance Liquid Chromatography (HPLC) method. Our study set barbiturate as the internal standard, a reversed-phase column was Column C18 (150×4.6mm, 5um) of DIKMA company, the mobile phase was methanol and water(40:60), at a flow rate of 1 ml/min, the detected wavelength was 215nm. Compared with homogeneous enzymatic amplification immunoassay, the HPLC method is time-consuming and easily interfered by sample pretreatment process. However, there is no homogeneous enzymatic amplification immunoassay method for the determination of LTG in China at present, and the methodological evaluation of LTG serum concentration determination had been well validated prior to the development of serum concentration monitoring.

### Group assignments

The patients were stratified into 3 groups according to the UN’s World Health Organization’s (WHO) definition of age, 3–14 years old population belong to children, 14–45 years old belong to youth and 45–59 years old belong to middle age. The patients were classified into 6 groups according to their concomitant AEDs regimens (Table 1): group A received LTG monotherapy, group B received LTG + AEDs with inducer, group C received LTG + valproic acid (VPA), group D received LTG + AEDs with non-inducers, group E received LTG + VPA + one or two AEDs with inducers, group F received LTG + VPA + AEDs with non-inducers.

**Table 1:** Patients and serum characteristics.

		Significance (P)
Number of samples	226	
Gender		
Male	110	
Female	116	
Age (years)		
Mean±SD (years)	22.94±12.66	
3–14	55	
14–45	150	
45–59	21	
LTG dose (mg/day)	158.50±52.38	
LTG dose/weight (mg/kg/day)	3.06±1.13	
LTG serum concentration (μg/ml)		
Mean±SD	5.48±3.83	
Male	5.60±3.81	0.64
Female	5.36±3.85	
3–14	4.99±3.55	0.05
14–45	5.88±3.94	
45–59	3.92±3.32	
Type of concomitant AEDs(number of samples)		
Group A: LTG monotherapy	71	
Group B: LTG+AEDs with inducer CBZ as the only concomitant AED OXC as the only concomitant AED TPM as the only concomitant AED CBZ + TPM as the concomitant AEDs	39 10 14 10 5	
Group C: LTG+VPA	68	
Group D: LTG+AEDs with non-inducer	11	
Group E: LTG+VPA+ AEDs with inducer	15	
Group F: LTG+VPA+AEDs with non-inducer	22	

Abbreviation:

AEDs: Antiepileptic drugs

LTG: Lamotrigine

CBZ: Carbamazepine

OXC: Oxcarbazepine

VPA: Valproic acid

TPM: Topiramate

### Statistical analysis

The results of continuous data were presented using their means, standard deviations (SD) (mean ± SD), the results of binary data were presented using percentages. Mean LTG serum concentrations were calculated according to different genders, ages, and types of concomitant AEDs using the descriptive statistical methods. To examine the differences of LTG serum concentration between genders, independent-sample t-test was used. To compare the LTG serum concentration of different age groups(3–14 years old, 14–45 years old, 45–59 years old) and different concomitant AEDs regimen groups, the t test for one-way ANOVA analysis and Bonferroni and Tamhane were adopted. To examine the possible effects of several influencing factors on the LTG serum concentration, the stepwise multivariate regression analysis was used. All P values were 2 tailed and were considered statistically significant for P <0.05. All statistical analyses were conducted using IBM SPSS, version 22.0.

## Results

### Patients and serum characteristics

226 serum samples met the inclusion criteria and were evaluated. Gender stratification was 110 males and 116 females. The mean LTG serum concentration was 5.48±3.83 μg/mL, and the other features of the patients and serum were showed in Table 1.

### Comparison of the serum concentration in patients in different groups

There were no gender differences in LTG serum concentration by using independent-sample t-test (P = 0.64), and there were no significant effects by age on serum LTG concentration after age stratification(3-14 years old, 14–45 years old, 45–59 years old)using one-way ANOVA analysis (P = 0.05) (Table 1).

We calculated the LTG serum concentrations of different AEDs regimens and compared the LTG serum concentrations of different groups with group A using one-way ANOVA analysis, and the Tamhane test was adopted. Table 2 showed the LTG daily dose and the mean serum concentrations in different groups. Our results showed that the LTG daily dose in 6 groups had no significant differences, the mean LTG serum concentration in group B was significantly lower (P = 0.04), in group C and F were significantly higher, and the mean LTG serum concentration in group E was higher than group A by 46.34%, it meant that hepatic enzyme inducers such as CBZ only partially compensated for the inhibitory effect of VPA in triple combination. For details see attached Table 2.

**Table 2:** Mean LTG daily dose and concentrations in the different concomitant AEDs groups and their statistical significance versus patients in group A.

	Number of samples	LTG dose (mg/day)	Significance (P)	LTG concentration Mean±SD (μg/mL)	Significance (P)
Group A: LTG monotherapy	71	156.93±52.99	--	3.97±2.06	--
Group B: LTG+AEDs with inducer	39	168.60±40.84	1.00	2.75±1.93	0.04
Group C: LTG+VPA	68	156.62±59.32	1.00	7.70±4.22	0.00
Group D: LTG+AEDs with non-inducer	11	165.90±39.20	1.00	4.43±1.42	0.99
Group E: LTG+VPA+ AEDs with inducer	15	158.30±32.27	1.00	5.81±3.71	0.72
Group F: LTG+VPA+ AEDs with non-inducer	22	148.00±63.31	1.00	8.66±4.50	0.00

LTG: Lamotrigine; VPA: Valproic acid

Inducer include: CBZ: Carbamazepine; OXC: Oxcarbazepine; TPM: Topiramate

non-inducer include: Levetiracetam; Clonazepam

In the group B, we classified the patients into 4 groups according to the different inducers: CBZ, OXC, TPM and CBZ +TPM. Compared with group A, the mean LTG serum concentrations from patients received CBZ and CBZ +TPM were significantly lower, and the serum from patients received OXC and TPM revealed no significant differences, for details see attached Table 3.

**Table 3:** Mean LTG daily dose and concentrations in different inducer groups and their statistical significance versus patients in group A.

	Number of samples	LTG dose (mg/day)	Significance (P)	LTG concentration Mean±SD (μg/mL)	Significance (P)
Group A: LTG monotherapy	71	156.93±52.99	--	3.97±2.06	--
CBZ as the only concomitant AED	10	170.00±34.96	1.00	1.84±1.44	0.00
OXC as the only concomitant AED	14	171.40±48.89	1.00	3.11±1.80	0.74
TPM as the only concomitant AED	10	172.50±29.93	1.00	4.20±1.74	1.00
CBZ + TPM as the concomitant AEDs	5	150.00±53.03	1.00	0.64±0.33	0.00

AEDs: Antiepileptic drugs; LTG: Lamotrigine; CBZ: Carbamazepine; OXC: Oxcarbazepine; VPA: Valproic acid; TPM: Topiramate

### Results of multiple regression analysis

The probable factors influencing the LTG serum concentration were examined according to multiple regression analysis using the stepwise selection procedure. Multiple regression analysis showed that the daily LTG dose and co-administration of other AEDs statistically affected LTG serum concentration. More precisely, co-administration of other AEDs showed the highest effect on LTG concentration (Standard coefficient β = 0.393; P = 0.000), followed by daily dose (Standard coefficient β = 0.234; P = 0.000). The coefficient of determination (R^2^) was 0.197, and the variables of gender, age, and the daily dose/weight were eliminated by stepwise selection.

## Discussion

The analysis of the influence on LTG serum concentration in our data showed that there were no significant gender and age effects, but the LTG daily dose and co-administration of other AEDs statistically affected LTG serum concentration. Combination with enzyme-inducer AEDs, the mean steady-state LTG concentration could be decreased by 30.73% compared with LTG monotherapy. Among enzyme-inducer AEDs, particularly strong inducer CBZ could decrease the mean LTG concentration by 53.65%, but weak inducer AEDs such as OXC and TPM had no effect, VPA could increase the mean LTG concentration by 93.95%, and the inducer only partially compensated for the inhibitory effect of VPA in triple combination.

Our date showed that there were no age effects on LTG concentration, which was inconsistent with the reported publication[16–20]. It indicated that the age was an independent factor affecting the LTG concentration, the age affected the blood concentration of many traditional epilepsy drugs, the body’s clearance rate of LTG would change significantly with age. In our study, we excluded patients ≤ 3 years old and ≥ 60 years old, and the most children were older age children, so the pharmacokinetic characteristics were close to adults, which was the cause leading to the inconsistent result. On the other hand, our sample size was too small and genetic polymorphisms in different populations would be the other causes. Levetiracetam(LEV)is neither the cytochrome P450 (CYP) nor UGT enzymes, and the protein binding rate is low, so it has no inhibitory or inducing effects on various isoenzymes and its propensity to interact with other AEDs is very low [21]. Clonazepam (CZP) was proved to be a potent noncompetitive or “mixed-type” competitive inhibitor of catalytic activities mediated by CYP2B[22], and LTG is extensively metabolized in the liver by UGT1A4 and UGT2B7[4,5], there are no interactions between lamotrigine and the drugs metabolized by cytochrome P450, So LEV and CZP were considered as non- enzyme inducer or inhibitor in our study.

The LTG concentration was decreased 53.65% by CBZ, but not by OXC and TPM in our study. In the instruction of OXC, OXC and its active metabolite monohydroxy derivative (MHD) can inhibit CYP2C19 enzyme, induce CYP3A4 and CYP3A5 enzyme and only slightly induced UDP- glucuronosyl transferase. Ma, et al [23] reported that SCN1A, UGT2B7 and ABCC2 genetic polymorphisms were associated with OXC maintenance doses, it meant that there would be interaction between OXC and LTG because of the common metabolic enzyme UGT2B7. Flesch G[24] reported that MHD was only a weak inducer of uridine diphospate (UDP)-glucuronyltransferase (UDPGT) and therefore was unlikely to have an effect on drugs that were mainly eliminated by conjugation through the UDPGT enzymes (e.g. valproic acid and lamotrigine). Our data also showed that the mean LTG serum concentration from patients received LTG+OXC had no difference compared with LTG monotherapy. Topiramate (TPM) is a weak hepatic enzyme inducer. The influence of TPM on LTG concentrations is inconsistent: In one study, TPM reduced plasma LTG concentrations by 40–50% in four of seven patients[25], in other larger studies TPM was without effect[26,27], Our data showed that TPM didn’t affect the LTG serum concentration, this is because TPM inhibits only CYP2C19 substrates [28], but LTG is metabolized by UGT enzymes.

In our hospital, The regimen of LTG + VPA is the most common treatment, Taing et al [29] also pointed out that a combination of LTG and VPA was considered particularly efficacious clinically showed a supra-additive suppression of ED’s, it would be the most common treatment. Gidal et al [30] reported that VPA inhibited the clearance of LTG at a low dose (125 mg/d) and a very low concentration (6.5 μg/mL). Our study showed that VPA could increase the mean LTG concentration by 93.95%, this was in line with the study of lamotrigine and valproate pharmacokinetics interactions [31], LTG is principally metabolized by UGT 1A4 and UGT2B7 [4,5], the LTG-VPA interaction in vivo arises from inhibition of UGT2B7[5]. The influence of polymorphism of UDP-glucuronosyltransferases and drug transporters on Pharmacokinetics of lamotrigine and its metabolite N-2-glucuronide [32] showed that variability influenced by UGT2B7 in lamotrigine pharmacokinetics was large; genotyping for UGT2B7 might be useful in various clinical settings. But the influence of valproic acid and polymorphism on serum concentration of lamotrigine in Chinese epileptic patients [33,34]showed that UGT1A4 polymorphism had an effect on LTG concentration only with VPA co-administration. This controversial conclusion might be related to different ethnic groups. So, it is very necessary and important to study the pharmacokinetic characteristics in different ethnic populations. The lack of the effect of genetic polymorphisms on LTG serum concentration was the limitation of this study. The mean LTG serum concentration in group LTG+VPA+ AEDs with inducer was higher than group LTG alone by 46.34%, it meant that hepatic enzyme inducers such as CBZ only partially compensated for the inhibitory effect of VPA in triple combination in our study.

In conclusion, our study showed that co-administration of other AEDs was the most influencing factor on the LTG serum concentration, and the mechanism of interaction between drugs was the inducing or inhibiting effects on the metabolic enzyme UGT 1A4 and UGT2B7 of LTG. Among AEDs, enzyme inhibitor VPA showed the highest impact on LTG concentration, followed by enzyme inducer CBZ, and inducer only partially compensated the inhibitory effect of VPA in triple combination. Oxcarbazepine, Topiramate, Levetiracetam and Clonazepam had no effect. So it is need to adjust the LTG dose when VPA or CBZ is prescribed or discontinued in the treatment regimen.

## Supporting information

S1 File The patient records. (XLS)

S2 File SPSS data. (XLS)
